# Helicopter Emergency Medical Service and Hospital Treatment Levels Affect Survival in Pediatric Trauma Patients

**DOI:** 10.3390/jcm10040837

**Published:** 2021-02-18

**Authors:** Felix Marius Bläsius, Klemens Horst, Jörg Christian Brokmann, Rolf Lefering, Hagen Andruszkow, Frank Hildebrand, TraumaRegister DGU®

**Affiliations:** 1 Department of Trauma and Reconstructive Surgery, University Hospital RWTH, 52074 Aachen, Germany; khorst@ukaachen.de (K.H.); handruszkow@ukaachen.de (H.A.); fhildebrand@ukaachen.de (F.H.); 2 Emergency Department, University Hospital RTWH, 52074 Aachen, Germany; jbrokmann@ukaachen.de; 3 Institute for Research in Operative Medicine (IFOM), Faculty of Health, Witten/Herdecke University, 51109 Cologne, Germany; Rolf.Lefering@uni-wh.de; 4 TraumaRegister DGU®, Academy for Trauma Surgery, Register and Research Coordination, 50939 Cologne, Germany; support-tr@auc-online.de

**Keywords:** Severely injured, children, kids, GEMS, HEMS, helicopter, polytrauma, treatment level

## Abstract

(1) Background: Data on the effects of helicopter emergency medical service (HEMS) transport and treatment on the survival of severely injured pediatric patients in high-level trauma centers remain unclear. (2) Methods: A national dataset from the TraumaRegister DGU^®^ was used to retrospectively compare the mortality rates among severely injured pediatric patients (1–15 years) who were transported by HEMS to those transported by ground emergency medical service (GEMS) and treated at trauma centers of different treatment levels (levels I–III). (3) Results: In total, 2755 pediatric trauma patients (age: 9.0 ± 4.8 years) were included in this study over five years. Transportation by HEMS resulted in a significant survival benefit compared to GEMS (odds ratio (OR) 0.489; 95% confidence interval (CI): 0.282–0.850). Pediatric trauma patients treated in level II or III trauma centers showed 34% and fourfold higher in-hospital mortality risk than those in level I trauma centers (level II: OR 1.34, 95% CI: 0.70–2.56; level III: OR 4.63, 95% CI: 1.33–16.09). (4) Conclusions: In our national pediatric trauma cohort, both HEMS transportation and treatment in level I trauma centers were independent factors of improved survival in pediatric trauma patients.

## 1. Introduction

Serious injuries remain the leading cause of death in children and adolescents [1]. Over the last decades, enormous efforts have been made to identify the risk factors associated with the high mortality rates among severely injured pediatric patients. Prehospital care and treatment levels of trauma centers have been widely reported as potential factors that can affect this outcome [2,3,4].

The potential advantages of helicopter emergency medical service (HEMS) for the prehospital care of trauma patients are still controversial in the literature. While the extensive experience of a HEMS crew and rapid transport to a suitable trauma center might be beneficial [4,5,6], factors such as high cost and limited availability (night time, severe weather conditions, etc.) raise concerns about the benefits of HEMS [7]. Data on pediatric trauma are even more sparse and inconsistent, and only a few studies with small sample sizes have investigated the relevance of HEMS transport in pediatric trauma. Even more, only a few studies have applied low or no Injury Severity Score (ISS) thresholds to determine the factors contributing to the survival of injured pediatric trauma patients [8,9]. Thus, these studies do not reflect the specific situation of severely injured children. In addition, most of the studies were conducted in the 1990s, hence recent developments in trauma care were not considered.

Concerning trauma center treatment levels, the results of non-European studies have indicated that (1) multidisciplinary trauma teams with pediatric commitment and capabilities, (2) the American College of Surgeons Committee on Trauma–verified centers, and (3) high-volume trauma centers may increase the survival rates of injured children [10,11]. However, these studies did not apply ISS thresholds and are therefore confounded, as they did not adjust for injury severity. Furthermore, the aforementioned studies can only partly reflect the current situation in European trauma systems due to differences between prehospital rescue systems (e.g., physician-staffed vs. paramedics) and clinical treatment strategies.

Addressing the clinical imperative of lowering the risk of death requires identifying the beneficial effects of preclinical parameters (e.g., transportation mode), as well as the influence of possible structural differences due to treatment at a high-level trauma center. By analyzing data from Germany, there is a unique opportunity to investigate the independent effect of the transport mode since both ground emergency medical service (GEMS) and HEMS are physician-staffed. Therefore, we designed this study to investigate our primary hypothesis that HEMS is associated with improved survival in severely injured children and the second hypothesis that treatment in a high-level trauma center is associated with a decreased mortality risk. To demonstrate the independent survival benefits of both parameters, we used a multivariate analysis and adjusted the observed mortality rates according to Revised Injury Severity Classification version 2 (RISC II) prognosis scores [12].

## 2. Materials and Methods

The TraumaRegister DGU^®^ (TR-DGU) of the German Trauma Society was founded in 1993 to create a multicenter database for the pseudonymized and standardized documentation of severely injured patients for quality assurance and research [13]. The participating hospitals are primarily located in Germany (90%), but a rising number of hospitals from other countries have also begun to contribute their data (e.g., Austria, Belgium, Finland). Currently, approximately 33,000 cases from more than 650 hospitals are entered into the database every year. Participation in the TR-DGU is voluntary; however, associated hospitals are obligated to enter at least one basic dataset for quality assurance purposes.

Documentation in the TR-DGU included detailed information on demographics, injury pattern, comorbidities, pre- and in-hospital management, course of care while in the intensive care unit, relevant laboratory findings (including data on transfusion), and the clinical outcome of each individual. The inclusion criteria for the TR-DGU were admission to a hospital via the emergency room with subsequent intensive care or entrance to a hospital with vital signs and death before admission to the intensive care unit (ICU).

The infrastructure for documentation and data management is provided by the Academy for Trauma Surgery (Akademie der Unfallchirurgie GmbH), a company affiliated to the German Trauma Society. Scientific leadership is provided by the Committee on Emergency Medicine, Intensive Care and Trauma Management (Sektion NIS) of the German Trauma Society. Scientific data analysis is approved according to a peer-review procedure laid down in the publication guideline of TR-DGU. The present study is consistent with the publication guideline of the TR-DGU and is registered as the TR-DGU project ID 2018-044.

### 2.1. Trauma Care in Germany

A nationwide helicopter rescue system was introduced in Germany in the early 1970s [7]. This system has developed into a modern HEMS, with a total of 89 helicopters nationwide and a complete air rescue coverage throughout the country. Both, HEMS and GEMS are physician-staffed, where the GEMS system is based on a rendez-vous system. The emergency physicians (HEMS and GEMS crews) are highly qualified and have undergone additional emergency physician training and international courses, such as the Prehospital Trauma Life Support course [14,15]. The vast majority (68%) of these emergency physicians are anesthetists and internists. Surgeons make up only about 13% of the emergency physicians [16]. An overview of the capabilities of the respective hospital care level is provided in Table S1.

### 2.2. Inclusion Criteria

**Figure 1:**
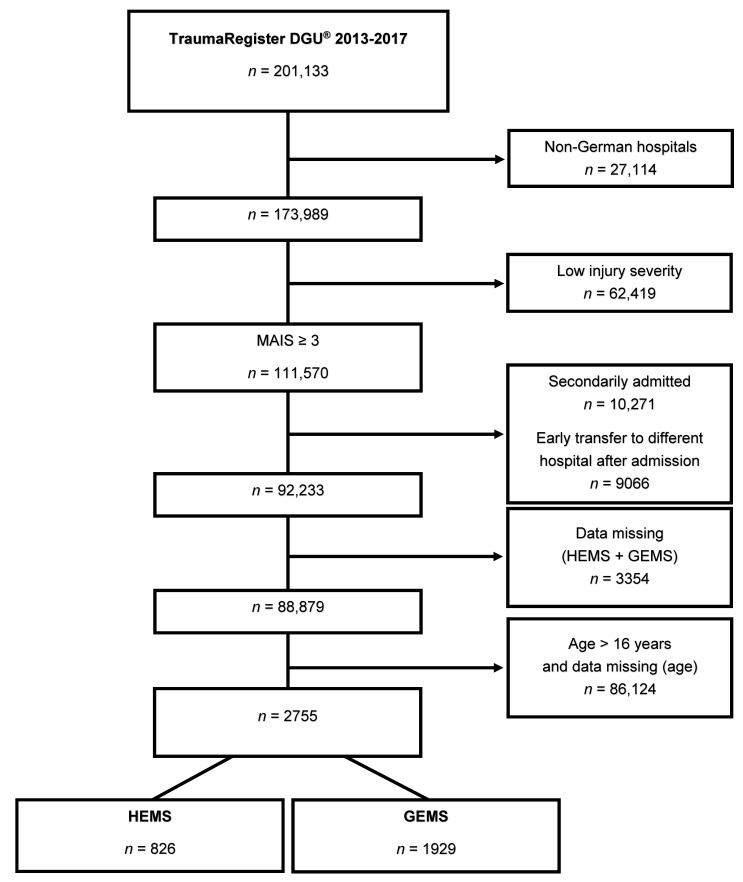
Study flow chart illustrating the selection of patients. MAIS: Maximum Abbreviated Injury Scale; HEMS: Helicopter Emergency Medical Service; GEMS: Ground Emergency Medical Service.

The inclusion and exclusion criteria are displayed in Figure 1: Flow chart.

### 2.3. Definitions

Patients younger than 16 years old were defined as children. The Abbreviated Injury Scale (AIS, Version 2005/Update 2008, Association for the Advancement of Automotive Medicine, Barrington, IL, USA) was adopted as the global system for injury severity determination. The severity of injuries was recorded according to the AIS as 1 (minor), 2 (moderate), 3 (severe, not life-threatening), 4 (serious, life-threatening), 5 (critical, survival uncertain), and 6 (maximum, currently untreatable). The maximum AIS (MAIS) was calculated for each patient according to the 2008 AIS Codebook Revision. The overall injury severity was calculated by ISS as described by Baker et al. [17].

### 2.4. Assessment of Mortality Risk

The RISC II score was developed and validated to predict the risk of death based on the TR-DGU data [12]. The score has 13 variables, which include injury pattern (AIS of the worst and second-worst injury), AIS_head_, Age, Gender, American Society of Anesthesiologists physical status classification system, motor function, pupil status (pupil reactivity and size), the injury mechanism, systolic blood pressure, coagulation (INR), acidosis (base deficit), hemoglobin levels, and CPR. RISC II scores were used to adjust the observed mortality rates by calculating the ratio of the observed to the expected mortality rate (standardized mortality ratio (SMR)). SMR values were given at 95% confidence intervals (CI) based on the respective CI of the observed mortality. The Student t-test was used to evaluate the differences in the SMRs. The RISC II scores are based on data from 2010 to 2011 and were therefore considered more suitable than the Trauma Injury Severity Score and the RISC II scores, which are based on older databases.

### 2.5. Statistics

To adjust for confounding variables, multivariable regression analysis with in-hospital mortality as the dependent endpoint was performed. Besides the mode of transportation, the hospital level of care (levels I–III) and RISC II scores, as a summary of patient-related factors, were considered confounders in the model. Since the treatment level and the transport mode are both structural factors that interact with each other, we included both in our analysis and could thus exclude the bias that severely injured children are predominately transported by HEMS to level I centers, which could have falsified our results. A final logistic regression analysis with hospital outcome as a dependent variable added the mode of transportation (ground and helicopter) and the level of care of the receiving hospital (local, regional, and supraregional) as additional predictors besides the RISC II score. The results were reported as odds ratios (ORs) at a 95% CI. In order to evaluate if a higher GEMS transportation rate during night time might have influenced out results, we added the interaction term night time x GEMS in a second model. Furthermore, we performed a subgroup analysis after excluding 20 highly unstable pediatric patients who died within 30 min of admission to avoid a bias regarding a higher OR in small level III trauma centers. We applied the threshold because it is regularly used to distinguish between CPR and prolonged CPR [18].

Categorical variables are presented as percentages only if the underlying total is obvious. Continuous values are presented as mean and standard deviation and median as interquartile ranges (IQRs 25–75), if applicable. Differences in categorical and continuous variables were evaluated with Pearson’s X^2^ test and the Wilcoxon rank-sum test, respectively. A two-sided *p*-value of less than 0.05 was considered statistically significant. The interpretation of the results generally focused on the clinical relevance of a difference rather than on *p*-values alone. The RISC II score was developed and validated using TR-DGU data and represents a summary of the 13 variables, including pattern and severity of injuries, age, sex, prior diseases, and initial physiology. Nagelkerke’s R² was used to describe the increase in the predictive power of the model. All statistical analyses were performed using the Statistical Package for Social Sciences (SPSS 25.0; IBM Inc., Armonk, NY, USA).

## 3. Results

### 3.1. Demographic Data

A total of 2755 pediatric trauma patients were included in the study (Figure 1). The mean age of the pediatric patients was 9.0 ± 4.8 years, and 63.5% were males. The proportion of pediatric trauma patients transported by HEMS was 30.0% (HEMS: 826 patients; GEMS: 1929 patients) (Table 1).

### 3.2. Cause of Injury, Injury Distribution, and Injury Severity

Overall, 83% of the missions took place during day time. Only 17% of all missions were performed at night time. During the night, the proportion of HEMS transports decreased from 32% to 20%. Traffic collision with pedestrians was the main accident in HEMS- and GEMS-transported pediatric trauma patients, with a significantly higher proportion in the GEMS population (24.4% vs. 19.6%; *p* = 0.006; Table 1). GEMS-transported children also sustained more low-fall (<3 m) accidents (14.9% vs. 8.6%; *p* < 0.001) and less frequent traffic accidents by car or bicycle (11.9% vs. 17.2%; *p* < 0.001; 14.0% vs. 18.6%, *p* = 0.002) than HEMS-transported children. 

The majority of the pediatric trauma patients were treated in level I (71.9%) and level II trauma centers (22.6%). HEMS-transported children had significantly higher ISS and increased incidence of severe head and chest injuries (Table 1). The presence of a traumatic brain injury was higher among patients transported with HEMS. Mission times and on scene treatment are displayed in Table 2.

### 3.3. Outcome

With an SMR of 0.87 (95% CI: 0.66–1.07), the observed mortality was lower than expected after HEMS transport, whereas the observed mortality was slightly higher after transport with GEMS (SMR: 1.11, 95% CI: 0.94–1.28) (Table 3).

After adjusting for the 13 variables included in the RISC II score and the hospital level of care, multivariable logistic regression analysis revealed that the OR for mortality among HEMS patients was 0.489 (95% CI: 0.282–0.850), showing significantly improved outcome benefits compared to GEMS (*p* = 0.011). Interestingly, with level I centers as the reference, the OR for mortality in level II and III trauma centers was 1.341 (95% CI: 0.702–2.564) and 4.625 (95% CI: 1.330–16.086), respectively (Table 4, model 1). Model 2 shows that after adding the interaction term night time X GEMS, HEMS transportation still shows an OR of 0.603, but the outcome difference is no longer significant. (Table 4, model 2).

If the hospital treatment level and mode of transportation were added to the 13 variables used in the RISC II (R^2^ = 0.595), the overall association with the outcome using Nagelkerke’s R^2^ reached 0.748 among the pediatric patients. This shows that by adding both variables to the 13 variables of the RISC II score, a notable stronger association of model 2 with the observed outcome (survival until discharge) could be achieved (Table 4).

If early deaths (up to 30 min after admission) were excluded, the effect for HEMS was 0.620 (95% CI: 0.337–1.139, *p* = 0.124)

## 4. Discussion

The potential benefits of HEMS for the preclinical treatment of pediatric trauma patients are still controversial in the literature. This disagreement might be explained by the limited comparability of different preclinical systems (physician- vs. paramedic-based) and a lack of focus on severely injured pediatric patients [19,20]. Besides the mode of transportation, the level of care is also assumed to be of major relevance in the outcomes of the pediatric trauma population [2,11].

In our study, HEMS transport was associated with a 50% reduction in the adjusted mortality risk (model 1). Brown et al. reported similar survival benefits in a matched-pair analysis of 17,657 helicopter transports of pediatric trauma patients from the National Trauma Data Bank in the US [8]. The authors adjusted for in-hospital confounders and found that one life may be saved for every 41 children transported by HEMS. In the 1990s, Moront et al. conducted a retrospective study among 3861 pediatric trauma patients admitted to level I trauma centers [21]; although the authors did not calculate the SMR and the group used the Trauma Injury Severity Score method, a cautious comparison can be made between their work and the present study. The authors claimed that 1.1 pediatric trauma patients were saved for every 100 transported by HEMS. This benefit was smaller than reported in our study and Brown et al.’s. Both aforementioned studies from the US included all hospital admissions during the study periods without ISS thresholds, and the study populations, therefore, showed considerably lower ISSs (Brown et al.: 58%, ISS < 9; Moront et al.: ISS 7.4 ± 1) than our study (ISS 19.1 ± 11.5). Brown et al.’s study also considered only long-lasting transports of more than 15 min, so the effects of HEMS missions within approximately 30 km (18.6 miles) of trauma centers were not investigated. However, these distances are crucial in urban and suburban areas, such as those found throughout Western Europe [22]. Since we strictly focused on severely injured patients (MAIS ≥ 3 and mean ISS 19), corrected for hospital treatment levels and the mode of transportation, and did not include limitations on transportation time, we are convinced that our study reflects, for the first time, the fundamental benefit of HEMS on survival after severe pediatric trauma. Additionally, due to the high injury severity in our study, the well-described rate of over-triage reported in previous studies for HEMS-transported pediatric trauma patients and the associated over-triage bias is likely reduced [21,23,24].

The aforementioned HEMS transportation-based survival benefit seems to be multifactorial. In our study, the difference in outcome appears to be largely due to the higher proportion of GEMS transports at night. Although, this effect itself shows no independent effect on mortality (model 2). Furthermore, HEMS staff are highly experienced in preclinical interventions and numerous studies have evinced that the rate of misintubation of pediatric patients is lowest when performed by HEMS personnel, especially if a physician is present [20,25,26]. In addition, rescue helicopters were introduced in Germany primarily for trauma care in order to shorten the time from accident to surgical therapy in the sense of the golden hour. Therefore, the relative proportion of trauma patients is higher in HEMS than in GEMS and could thus contribute to greater experience of HEMS crews [7]. A low intervention threshold is also responsible for the higher intervention rates of HEMS crews, as these measures cannot be carried out during flights and stopovers should be avoided, if possible. At least, in our study, the relatively high injury severity of HEMS patients was also likely to result partly in the greater necessity of interventions (intubation, sedation, and volume resuscitation). Prolonged on-scene treatment is obviously one reason for longer prehospital times in HEMS-treated patients in both our studies and other studies (Table 2) [6]. However, other reasons exist for this prolonged period before hospital admission. In this context, HEMS is frequently used for patient transportation after initial treatment by GEMS (40% of HEMS transports) [27]. For this reason, the mission times of HEMS are likely biased due to many secondary call-outs.

Another reason for the beneficial effects of HEMS transport in pediatric patients appears to be shortened transport time to level I trauma centers and the availability of highly experienced trauma teams. Using these level I trauma centers as a reference, we found a four-fold higher adjusted mortality risk for pediatric patients treated in level III trauma centers. Even level II centers were associated with a 34% increase in mortality risk compared to level I centers. The survival benefit of level I center treatment compared to a level III treatment was even higher than the independently observed survival benefit of HEMS transportation. This is consistent with the findings of Miyata et al., which showed survival benefits for pediatric trauma patients in high-volume trauma centers in a cohort of over 3700 children with ISS greater than 25 [28]. These results have been confirmed by several studies, underlining the survival benefits of specialized pediatric trauma centers [10,11,29,30]. Interestingly, Brown et al. included in-hospital confounders, such as trauma center type, and demonstrated a two-fold increase in survival for treatment in level I compared to level II pediatric trauma centers [8]. However, no survival benefit was found when only the levels of care were compared without considering the pediatric experience in the trauma center. Thus, Brown et al.’s study supports the recommendation to reduce mortality by incorporating pediatric experience into the trauma care of severely injured children. Consistent with our study, Biewener et al. claimed that hospital treatment levels had a higher impact on in-hospital mortality than transportation mode. However, due to their methodology, the authors could not make a final statement on the independent effects of HEMS transport and level of care [4]. In our study, we adjusted the observed mortality rates using RISC II and included the transportation mode and the level of care as structural confounders. Thus, we were able to consider the influence of these structural variables independently of each other.

In summary, our data and the results of previous studies support the treatment of severely injured children in level I centers to improve treatment outcomes after major trauma. As severe pediatric trauma per se is a relatively rare event, a lack of exposure and infrastructure in level III trauma centers is therefore likely. Therefore, the experience and expertise of specialized trauma teams, including pediatricians, as well as established treatment protocols for pediatric trauma patients, presumably resulted in shorter resuscitation times, faster diagnostic procedures, and reduced periods until surgical treatment in high-volume centers, thereby improving outcomes. Furthermore, the capabilities of these centers helped to avoid secondary transfers. Based on these results, every regional TraumaNetzwerk DGU^®^ in Germany should maintain at least one pediatric trauma referral center that fulfills diverse prerequisites, such as medical personnel with specific pediatric trauma competence, child-specific treatment protocols, and a pediatric intensive care unit (Table S1) [31].

Although we confirmed the results of previous studies that described the beneficial effects of HEMS transport and treatment in high-volume, high-level centers for adults [32,33], we also found that these factors had an even more substantial impact on survival in children than previously thought. Besides differences in etiology, injury patterns, and physiology, the rarity of pediatric trauma means that experience treating such patients (including specific treatment protocols, pediatric ICU, etc.) has an enormous effect on the outcomes of severely injured children [34]. Therefore, interdisciplinary efforts to provide trauma care for severely injured children with pediatricians and high-level treatment to respond to the specificities of pediatric patients must be strongly supported.

### Strength and Limitations

Beside the focus on severely injured patients (MAIS ≥ 3), another strength of our study is the exclusion of patients who died within 30 min of admission in a subgroup analysis, avoiding potential bias, as these unstable, untransportable patients might have been transported to the nearest level III trauma centers. Since the results remained comparable, these patients surely did not significantly influence our results. Finally, we included both trauma center level and transport mode in our multivariable analysis to exclude the correlation between HEMS transportation and a higher admission rate to level I and II trauma centers.

Regarding the limitations, our multivariable regression model did not rule out all confounders that were not considered. Our study was also limited by its retrospective study design and reliance on accurate individual records within the registry, although the data quality of the TR-DGU is generally considered high [13].

## 5. Conclusions

We observed a survival benefit for HEMS-transported severely injured pediatric patients as well as an independent 34% and four-fold higher mortality risk for pediatric patients treated in level II and III trauma centers, respectively. Based on these observations, triage criteria should be developed to identify children who should be transported by HEMS and treated in high-level trauma centers by interdisciplinary teams with pediatric experience to increase the survival of severely injured children.

## Figures and Tables

**Table 1:** Cause of injury, injury distribution and injury severity of the pediatric study population.

	HEMS	GEMS	*p*-Value
Proportion (%)	30.0	70.0	
Age ± SD (years)	9.0 ± 4.7	9.1 ± 4.8	0.45
Car accident (%)	17.2	11.9	<0.001
Motorcycle accident (%)	5.3	5.0	0.72
Bicycle accident (%)	18.6	14.0	0.002
Pedestrian traffic accident (%)	19.6	24.4	0.006
Height fall > 3 m (%)	17.8	16.9	0.55
Height fall < 3 m (%)	8.6	14.9	<0.001
Others (%)	12.9	12.9	0.96
ISS ± SD (pts.)	21.4 ± 12.6	18.1 ± 10.8	<0.001
AIS_Head_ ≥ 3 (%)	60.3	46.9	<0.001
AIS_Thorax_ ≥ 3 (%)	30.6	25.4	0.005
AIS_Abdomen_ ≥ 3 (%)	10.8	13.9	0.026
AIS_Extremity_ ≥ 3 (%)	30.1	33.4	0.096
Injury pattern			<0.001
Head only (%)	22.5	21.3	
Combined (%)	46.0	31.9	
Body only (%)	31.5	46.8	
Systolic blood pressure < 90mmHg—pre-hospital (%)	14.9	16.4	0.39
Systolic blood pressure < 90mmHg—ER (%)	12.6	11.3	0.35
GCS < 9 pts. (%)	33.0	20.0	<0.001
Night time (%)	11.4	19.4	<0.001

HEMS: Helicopter Emergency Medical Service; GEMS: Ground Emergency Medical Service. ISS: Injury Severity Score; AIS: Abbreviated Injury Scale; GCS: Glasgow Coma Scale; ER: Emergency Room.

**Table 2:** Mission times and on-scene treatment in the pediatric study group.

	HEMS	GEMS	*n*	*p*-Value
Transportation time from scene to hospital (min)	20.4 ± 12.3	17.8 ± 11.3	983	<0.001
Total time—from injury to hospital (min)	72.9 ± 27.1	55.7 ± 25.0	2177	<0.001
Intubation (%)	52.3	21.7	2670	<0.001
Vasopressors (%)	9.8	7.4	1498	0.096
Chest tube (%)	1.8	0.9	1498	0.15
CPR (%)	4.9	5.7	2670	0.38
Sedation (%)	76.3	61.9	1498	<0.001
Volume resuscitation (%)	90.2	78.7	2510	<0.001

CPR: Cardiopulmonary Resuscitation.

**Table 3:** Survival benefit of helicopter emergency medical service (HEMS) measured by Revised Injury Severity Classification version 2 (RISC II) in the pediatric study group.

	HEMS	GEMS	*p*-Value
Number of cases	826	1929	
Expected mortality	9.1%	7.0%	<0.001
Observed mortality	7.9%	7.8%	0.93
Standardized Mortality Ratio (SMR) (95% CI)	0.87 (0.66 to 1.07)	1.11 (0.94 to 1.28)	0.099

**Table 4:** Odds ratios for parameters that showed independent association with the mortality in multivariable analysis for the pediatric study group (Model 1) and after adding the interaction term night time X ground emergency medical service (GEMS) (Model 2). RISC II: Revised Injury Severity Score.

***Model 1***	**Odds Ratio**	**95% CI**	***p*** **-Value**
RISC II	0.309	0.27–0.353	<0.001
Level I trauma centers (reference)			0.048
Level II	1.341	0.702–2.564	0.374
Level III	4.625	1.330–16.086	0.016
Transportation: HEMS	0.489	0.282–0.850	0.011
***Model 2***			
RISC II	0.308	0.269–0.352	<0.001
Level I trauma centers(reference)			0.032
Level II	1.400	0.730–2.680	0.311
Level III	5.184	1.461–18.38	0.016
Transportation: HEMS	0.603	0.332–1.094	0.012
Interaction night time X GEMS	1.873	0.973–3.468	0.061

## Data Availability

Data are provided by the TraumaRegister DGU^®^. Data are available from the TraumaRegister DGU^®^ for researchers who meet the criteria for access to confidential data.

## Supplementary Materials

The following are available online at https://www.mdpi.com/2077-0383/10/4/837/s1, Table S1: Trauma care in Germany and trauma center specifications.

## Abbreviations

AIS	Abbreviated Injury Scale
AUC	Academy for Trauma Surgery
DGU	German Trauma Society
GEMS	Ground Emergency Medical Services
HEMS	Helicopter Emergency Medical Services
ICU	Intensive Care Unit
ICU LOS	Intensive Care Unit length of stay
IQR	Interquartile Range
ISS	Injury Severity Score
LOS	Length of stay
MAIS	Maximum AIS
OR	Odds ratio
RISC II	Revised Injury Severity Classification Score II
SD	Standard Deviation
SMR	Standardized Mortality Ratio
TR-DGU	TraumaRegister DGU^®^
