# Supplementary material for: Helicopter Emergency Medical Service and Hospital Treatment Levels Affect Survival in Pediatric Trauma Patients
Source: J Clin Med. 2021 Feb 18;10(4):837. doi: 10.3390/jcm10040837 (PMC7922049; doi:10.3390/jcm10040837)
Supplement: Supplementary file 1 [file jcm-10-00837-s001.pdf]

Supplement 1

Trauma care in Germany

The beginnings of structured emergency rescue in Germany can be traced back to the late 1920s. Of the initial two systems, which included either physician-staffed GEMS or a rendez-vous system, the rendez-vous system has now become the predominant rescue system.

TraumaNetzwerk DGU

- Founded in 2006
- To date 624 certified trauma centers have been certified and 52 regional trauma networks have been established
    - 109 level I – supraregional maximum care provider
    - 209 level II – regional trauma center
    - 306 level III – local trauma center

Level I - supraregional maximum care provider

- Clinic for trauma surgery or clinic for surgery with a trauma surgery section
- Round the clock: Specialist for trauma surgery with an additional training in special trauma surgery
- Round the clock: Specialist for visceral surgery or general surgery
- Round the clock: Specialist in anesthesiology
- Round the clock: Specialist in radiology
- Round the clock: Specialist in neurotraumatology
- Round the clock: Specialist in vascular surgery
- Round the clock: Availability of all specialities involved in the treatment of severely injured patients (e.g. pediatricians, maxillofacial surgery)

- Round the clock: Obligation to treat at least two seriously injured patients admitted within 1 hour
- Participation in prehospital emergency medical care
- Round the clock: Intensive care unit capacities for at least two severely injured patients
- Round the clock: Capacities for emergency operations

Level II - regional trauma center

- Clinic for trauma surgery or clinic for surgery with a trauma surgery section
- Round the clock: Specialist for trauma surgery with an additional training in special trauma surgery
- Round the clock: Specialist for visceral surgery or general surgery
- Round the clock: Specialist in anesthesiology
- Round the clock: Specialist in radiology
- Round the clock: Specialist in neurotraumatology
- Round the clock: Specialist in vascular surgery
- Round the clock: Obligation to treat at least one seriously injured patient
- Round the clock: Intensive care unit capacities for at least one severely injured patients
- Round the clock: Capacities for emergency operations

Level III - local trauma center

- Clinic for trauma surgery or clinic for surgery with a trauma surgery section
- Round the clock: Specialist for trauma surgery
- Round the clock: specialist for visceral surgery or general surgery
- Round the clock: Specialist in anesthesiology
- Round the clock: Capacities for emergency operations

Pediatric trauma referral center

- At least one center per regional trauma network
    - Usually a level I university hospital
- Pediatric intensive care unit
- Specific pediatric trauma competence (e.g. specialist in pediatric surgery)
- Child-specific treatment protocols

Whitebook Medical Care of the Severely Injured

The detailed specifications of each level of care are published in the *Whitebook Medical Care of the Severely Injured*.

GermanTraumaSociety. Whitebook Medical Care of the Severely Injured 2nd revised and updated edition. 2012;

https://www.dgu-online.de/fileadmin/published_content/5.Qualitaet_und_Sicherheit/PDF/2012_DGU_Whitebook_Medical_Care_2ndEdition.pdf.
